# Adult Human Biliary Tree Stem Cells Differentiate to β-Pancreatic Islet Cells by Treatment with a Recombinant Human Pdx1 Peptide

**DOI:** 10.1371/journal.pone.0134677

**Published:** 2015-08-07

**Authors:** Vincenzo Cardinale, Rosa Puca, Guido Carpino, Gaia Scafetta, Anastasia Renzi, Michele De Canio, Francesca Sicilia, Lorenzo Nevi, Domenico Casa, Rocco Panetta, Pasquale Bartolomeo Berloco, Lola M. Reid, Giorgio Federici, Eugenio Gaudio, Marella Maroder, Domenico Alvaro

**Affiliations:** 1 Department of Medico-Surgical Sciences and Biotechnologies, Sapienza University of Rome, Rome, Italy; 2 Department of Health Sciences, University of Rome “Foro Italico”, Rome, Italy; 3 Departments of Science and Chemical Technologies, University of Tor Vergata, Rome, Italy; 4 Italian Federation of Juvenile Diabetes (FDG), Rome, Italy; 5 Department of General Surgery and Organ Transplantation, Sapienza University of Rome, Rome, Italy; 6 Departments of Cell and Molecular Physiology, Program in Molecular Biology and Biotechnology, University of North Carolina School of Medicine, Chapel Hill, North Carolina, United States of America; 7 Department of Anatomical, Histological, Forensic Medicine and Orthopedics Sciences, Sapienza University of Rome, Rome, Italy; 8 Eleonora Lorillard Spencer-Cenci Foundation, Rome, Italy; Bambino Gesu' Children Hospital, ITALY

## Abstract

Generation of β-pancreatic cells represents a major goal in research. The aim of this study was to explore a protein-based strategy to induce differentiation of human biliary tree stem cells (hBTSCs) towards β-pancreatic cells. A plasmid containing the sequence of the human pancreatic and duodenal homeobox 1 (PDX1) has been expressed in E. coli. Epithelial-Cell-Adhesion-Molecule positive hBTSCs or mature human hepatocyte cell line, HepG2, were grown in medium to which Pdx1 peptide was added. Differentiation toward pancreatic islet cells were evaluated by the expression of the β-cell transcription factors, Pdx1 and musculoapo-neurotic fibrosarcoma oncogene homolog A, and of the pancreatic hormones, insulin, glucagon, and somatostatin, investigated by real time polymerase chain reaction, western blot, light microscopy and immunofluorescence. C-peptide secretion in response to high glucose was also measured. Results indicated how purified Pdx1 protein corresponding to the primary structure of the human Pdx1 by mass spectroscopy was efficiently produced in bacteria, and transduced into hBTSCs. Pdx1 exposure triggered the expression of both intermediate and mature stage β-cell differentiation markers only in hBTSCs but not in HepG2 cell line. Furthermore, hBTSCs exposed to Pdx1 showed up-regulation of insulin, glucagon and somatostatin genes and formation of 3-dimensional islet-like structures intensely positive for insulin and glucagon. Finally, Pdx1-induced islet-like structures exhibited glucose-regulated C-peptide secretion. In conclusion, the human Pdx1 is highly effective in triggering hBTSC differentiation toward functional β-pancreatic cells.

## Introduction

In the last years, several attempts to reprogram liver cells into pancreatic endocrine cells have been proposed [1–3]. Pdx1 or Pdx1-VP16 protein transduction, for example, induce β-cell gene expression in the rat hepatic cell line (WB-F344) with stem cell-like features [1]. Adult mouse intrahepatic cholangiocytes have been induced *in vitro* to become insulin-producing cells by transfection with adenoviral (Ad)-Pdx1 which induces not only insulin but also Glut2 and Prohormone convertase 1 and 2 expression [2]. Also populations of primary cells from surgically resected liver wedge of Yorkshire pigs, electroporated with an insulin expression plasmid, demonstrated functional pancreatic differentiation [3]. However, cells with different degree of hepatic maturation lineage showed large differences in the capability to achieve endocrine pancreatic differentiation. Indeed, the viral transfection of a single lineage transcription factor, Ngn3, induced cell-lineage switching from hepatic to an islet lineage only in progenitor cells but not in terminally-differentiated hepatocytes [4]. Moreover, in a recent study by Banga et al. [5] a strategy to drive liver cell toward pancreatic endocrine cells through genetic reprogramming of Pdx1 expression, culminated in the expression of pancreatic islet markers specifically within glandular Sox9 positive elements of the bile ducts. We have recently identified a heterogeneous stem/progenitor cell population within the peribiliary glands (PBGs) of the human biliary tree [6–11]. These cells, referred to as human Biliary Tree Stem/progenitor Cells (hBTSCs), express a broad panel of endoderm stem cell markers, display *in vitro* long-term persistence and self-renewal, and are able to give rise to a more restricted progeny of different mature lineages (hepatocytes, cholangiocytes and β-pancreatic cells) [6–11]. A number of limitations and drawbacks inherent to standard retroviral reprogramming methodology still persist (permanent genetic alterations) [12]. Therefore, we sought to induce the differentiation of hBTSCs to functional insulin-producing cells trough an innovative protein-based strategy.

## Materials and Methods

### Pdx1 production

Recombinant Pdx1 was obtained in the form of a fusion protein by linking 6His-tag to the N-terminus of amino acid sequence. Full-length DNA coding sequence for human PDX1 (852 bp coding for 283 aa) adapted for heterologous expression in *E*. *coli* was provided by GenScript USA Inc. (Piscataway, NJ). The sequence was amplified by PCR using primers 5’-TATCATATGAACGGTGAAGAACAGTACTAC-3’ and 5’-ATACTCGAGCTAACGTGGTTCTTGCGGACGGC-3’. After digestion with NdeI and BamHI, the amplicon was ligated into pET-28a expression vector (Novagen-Merck, Darmstadt, Germany), yielding pET-PDX1 plasmid. This construct was used to transform the BL21 (DE3) *E*. *coli* strain (Invitrogen, Italy).

### Pdx1 purification

The cellular extract was loaded on a 10 ml column of Ni^2+^-activated Chelating Sepharose FF (GE Heathcare, Italy). Fractions containing Pdx1 protein was analyzed by SDS-PAGE. A Sephadex G-25 column (GE Heathcare, Italy) was employed to remove imidazole and to exchange buffer with PBS. Mass spectrometry analyses were performed after tryptic digestion of the band of 43 kDa isolated by Coomassie blue stained gel. Mass spectra were acquired by Ultraflex III MALDI-TOF-TOF instrument (Bruker-Daltonics, Bremen, Germany), and peptide sites were searched in the NCBI database by MASCOT search engine.

### Isolation of hBTSC from human extrahepatic biliary tree

Normal adult human biliary tissues were collected from intact livers and pancreata obtained from organ donors at Sapienza University of Rome, Rome, Italy. We received the biliary tree from donor organs, since most of the biliary tree, comprising bile duct, cystic duct, gallbladder and common hepato-pancreatic ampulla, is routinely removed during liver transplantation procedures. The research protocol was reviewed and approved on 17 June 2010 (Protocol 541/10) by the *Ethics Committee of Hospital Policlinico Umberto I of Rome* (full name of the board/committee), and the processing was compliant with Good Manufacturing Practice. The written informed consent from the next of kin was obtained for the use of the sample for this research study. Tissue specimens were digested in RPMI 1640 supplemented with 0.1% bovine serum albumin, 1nM selenium, antibiotics, type I collagenase (300 collagen digestion unit/ml), 0.3 mg/ml deoxyribonuclease, at 37°C with frequent agitation for 30–45 min. Suspensions were filtered through a 500 micron metallic mesh filter (IDEALE ACLRI9 inox stainless steel) and spun at 1200 RPM for 5 min before resuspension of the cells. Thereafter, cell suspensions were passed consecutively through 100 and 30 micron mesh filter; then, cell counting was carried-out by Fast-Read 102 (Biosigma Srl, Venice, Italy) and cell viability (expressed as % of total cells) by the Trypan Blue exclusion assay. Cell viability was constantly higher than 95%. Isolated extrahepatic biliary cells were sorted for EpCAM+ by using EpCAM MicroBeads (Miltenyi Biotec Inc., catalog #130-061-101Germany) as indicated by the manufacturer. Then, the cell suspension was loaded onto a MACS LS Column (Miltenyi Biotec Inc., catalog #130-042-401) that was placed in the magnetic field of a MACS Separator. The magnetically labeled EpCAM+ cells were retained within the column, while the unlabeled cells passed through. After removing the column from the magnetic field, the magnetically retained EpCAM+ cells were eluted as a positively selected cell fraction. EpCAM+ cells were evaluated by cell count and cell viability as previous described.

### Control cells

Normal pancreatic islet cells were purchased by ProdoLab, Irvine CA US (HIR-001), human pancreatic beta cell line (PANC-1 hybrid cell line 1.1B4) was purchased by Sigma-Aldrich (cat. N.100128101), and were used as positive controls.

HepG2 cells, purchased commercially by ATCC No. HB-8065, are a suitable *in vitro* model to study polarized human hepatocytes; HepG2 cells were incubated with Pdx1 media and used as a putative negative control.

### Media and Solutions

Cells included in our experimental design were cultured in several conditions. All media were sterile-filtered (0.22-μm filter) and kept in the dark at 4°C before use. RPMI-1640, the basal medium for all cell cultures, and fetal bovine serum (FBS) were obtained from GIBCO/Invitrogen (Carlsbad, CA). All reagents were obtained from Sigma (St. Louis, MO) unless otherwise specified. Growth factors, except those noted, were purchased from R&D Systems (Minneapolis, MN).

- **Kubota’s Medium (KM)**. basal medium (here being RPMI 1640) with no copper, low calcium (0.3 mM), 10–9 M Selenium, 0.1% bovine serum albumin (BSA), 4.5 mM Nicotinamide, 0.1 nM Zinc Sulfate heptahydrate, 10–8 M hydrocortisone (or dexamethasone), 5 μg/ml transferrin/Fe, 5 μg/ml insulin, 10 μg/ml high density lipoprotein, and a mixture of free fatty acids that are added bound to purified human serum albumin. Mature epithelial cells of liver, biliary tree, and pancreas do not survive in this medium [13]. These conditions were found to be effective in further selecting EpCAM+ cells and determinating the disappearance of mesenchymal cells after 6–10 days.
- **Modified Kubota’s Medium (MKM)**. Serum-free KM without hydrocortisone was supplemented with calcium (final concentration 0.6 mM), copper (10–12 M) and 20 ng/ml bFGF [14, 15].
- **High-Defined Medium for Pancreatic islet cell differentiation (HDM-P)**. MKM, supplemented with 2% B27, 0.1 mM ascorbic acid, 0.25 μM cyclopamine, 1 μM retinoic acid; bFGF was added for the first 4 days and then replaced with 50 ng/ml exendin-4 and 20 ng/ml of HGF[14, 16].
- **Pdx1 media [0.1 or 0.5** μ**M].** MKM, supplemented with PDX-1 peptide [0.1 or 0.5 μM].

### *In vitro* differentiation of hBTSCs towards pancreatic fate

The capability of Pdx1 peptide to induce pancreatic differentiation in hBTSCs was tested *in vitro* as following. EpCAM+ hBTSCs were subjected to self-replication conditions in serum-free Kubota’s Medium (KM) and culture plastic. Self-replication culture conditions obtained by expansion in serum-free Kubota’s Medium (KM) resulted in the selection of a population of stem/progenitor cells positive for EpCAM and the disappearance of fibroblasts and mature epithelial cells from human liver and biliary tree [9, 11, 13, 14, 16, 17]. We have recently demonstrated [11, 17] that EpCAM+ cells, sorted from human adult biliary tree and subjected to expansion in serum-free KM, under same conditions adopted here, give rise to cell cultures that are composed by EpCAM positive cells (>95%) but rare cells of mesenchymal origin (<1%) [11]. Cultures were maintained for 6 days in serum-free KM. Thereafter, hBTSCs were incubated for further 14 days in:

- KM;
- HDM-P;
- Pdx1 medium [0.1 μM];
- Pdx1 medium [0.5 μM].

Accordingly, HepG2 cells, used as negative control, were incubated for 14 days in:

- KM;
- Pdx1 medium [0.1 μM];
- Pdx1 medium [0.5 μM].

Cell viability, after incubation with different Pdx-1 concentrations, was evaluated every 3 days by trypan blue exclusion test.

### Protein extraction and western blot (W-B) analysis

For protein analysis, cells were lysated in RIPA buffer plus protease inhibitors. 50 μg of proteins were loaded onto 12.5% polyacrylamide gel. Proteins were transferred onto PVDF membrane by electrophoresis. Once transferred, membranes were blocked in BSA 5% for 1 hour at Room Temperature (RT). Anti His-tag primary antibody (Cell Signaling Technology; cat#: 2366) was incubated at a 1:1000 diluition in 5% BSA overnight at 4°C. The membrane was then washed three times before the addition of secondary antibody (goat anti rabbit- HRP 1:10000) for 1 hour at RT.

### Reverse-transcription polymerase chain reaction (RT-PCR) analysis

Total RNA from cell cultures was extracted by the procedures of Chomczynski and Sacchi [18]. RNA was extracted by TRIZOL reagent (Life Technologies; Cat# 15596–026) according to the manufacturer’s instructions. 1 μg of RNA was retrotranscribed using High Capacity cDNA Reverse Transcription Kit (Applied Biosystem; cod. 4368814) and cDNA was amplified using SensiMix SYBR kit (Bioline, cod. QT605-05) according to the manufacturer ‘s instructions.

Primers used were: for *insulin* fw TATAAAGCTGGTGGGCATCC, rev GCCATGTTGAAACAATGAC; for *glucagon* fw AGCTGCCTTGTACCAGCATT, rev TGCTCTCTCTTCACCTGCTCT; for *somatostatin* fw TGGGTTCAGACAGCAGCTC, rev CCCAGACTCCGTCAGTTTCT; for *MafA* fw TTCTCCTTGTACAGGTCCCG, rev GAGAGCGAGAAGTGCCAACT; for *Pdx1* fw CATTGGAAGGCTCCCTAACA, rev TCCCACTGGCATCAATTTCA; for *EpCAM* ATAACCTGCTCTGAGCGAGTG, rev TGAAGTGCAGTCCGCAAACT; for *18S* fw GCAATTATTCCCCATGAACG, rev GGGACTTAATCAACGCAAGC. The expression of the gene of interest was calculated by the ratio of the concentrations of the gene of interest and the reference gene 18S.

### Measurement of C-peptide secretion in hBTSCs

Cells were washed three times with glucose-free RPMI 1640 (GIBCO, catalogue #11879) and pre-incubated for 12 hours in glucose-free RPMI 1640 supplemented with 5.5 mM glucose, 0.1% BSA, and antibiotics. After pre-incubation, medium was removed, and cells were gently washed three times with glucose-free RPMI 1640. Cells were incubated for additional 1 hour in glucose-free RPMI 1640 supplemented with 5.5 mM glucose and 0.1% BSA. Medium was collected and stored at -20°C. Cells were again gently washed three times with glucose-free RPMI 1640 and then incubated for 1 hour in glucose-free RPMI 1640 supplemented with 28 mM glucose and 0.1% BSA. Again, medium from each well was collected and stored at -20°C. Samples from cultures at 5.5 mM versus 28 mM glucose were used for assays of C-peptide synthesis. After media collection, the cells were collected separately from each well and counted using the Trypan blue exclusion assay. The human C-peptide content in the medium was measured by an ELISA kit (Mercodia cod. N. 10-1136-01; Uppsala, Sweden) and normalized to the cell number of each sample. The amount of C-peptide generated in response to the high-glucose challenge was divided by the amount generated by the low-glucose challenge to yield the mean C-peptidesecretion index. The stimulation index of C-peptide secretion is calculated as the ratio between C-peptide secreted in the medium under high glucose concentration and C-peptide secreted under basal (low) glucose concentration; C-peptide concentration in the medium was quantified by ELISA in the same cell sample and during a fixed time period (1 h).

### Light Microscopy (LM) and Immunofluorescence (IF)

Cells cultured in chamber slides were fixed in acetone for 10 minutes. Fixed cells were incubated with primary antibodies (Guinea pig anti-human insulin, DAKO, cat#IS002, dilution: 1:100; Rabbit anti-human glucagon, Santa Cruz Biotechnologies, cat# SC-13091, dilution: 1:50). Then, cells were washed and incubated for 1h with labeled isotype-specific secondary antibodies (anti-guinea pig AlexaFluor-488, anti-rabbit Alexafluor-546, Invitrogen, Life Technologies Ltd, Paisley, UK) and counterstained with 4,6-diamidino-2-phenylindole (DAPI) for visualization of cell nuclei. For all immunoreactions, adequate positive controls were performed. Slides were examined in a coded fashion using a Leica Microsystems DM 4500 B Light and Fluorescence Microscopy (Weltzlar, Germany) equipped with a Jenoptik Prog Res C10 Plus Videocam (Jena, Germany). IF staining was also analyzed by Confocal Microscopy (Leica TCS-SP2).

Slides have been further observed by phase contrast microscope. The number of islet-like structures (insulin-positive) was counted in a random, blinded fashion in three non-overlapping fields (magnification x20) for each chamber slide; at least, 5 chamber slides have been examined for each treatment.

### Statistical Analysis

Data were expressed as mean ± standard deviation (SD). Statistical analyses were performed by SPSS statistical software (SPSS Inc. Chicago IL, USA). Differences between groups for non-normal distribution parameters were tested by Mann–Whitney U tests. Statistical significance was set to a *p*-value < 0.05.

## Results

### Pdx1 production

**Fig 1:**
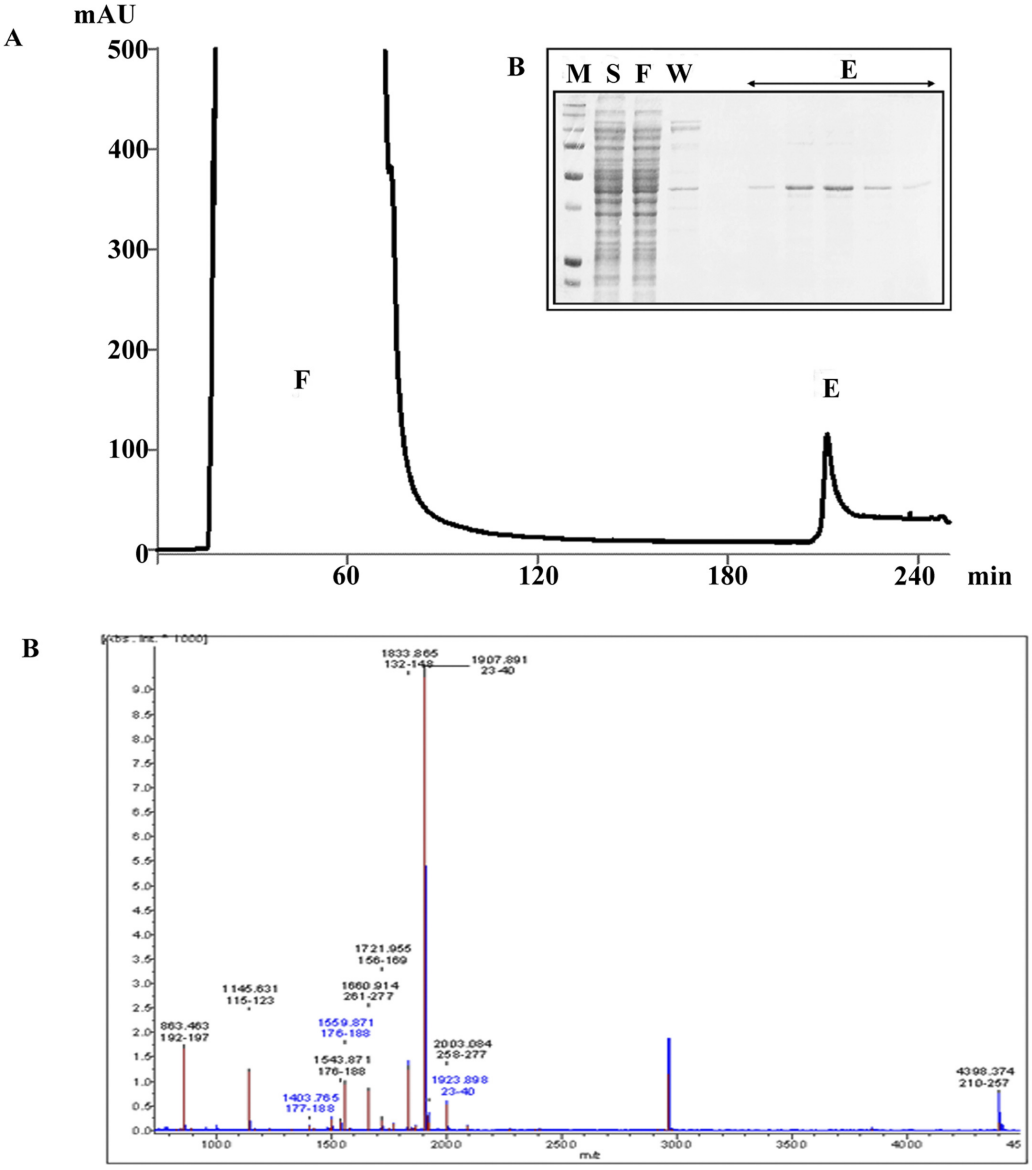
Nickel Chelating Sepharose chromatography of recombinant 6His-tagged Pdx1 and identity of purified Pdx1 protein by Mass Spectrometry. (A) Clarified bacterial lysate was applied to a 10 mL column of Chelating Sepharose activated with Ni^2+^ ions. Chromatographic steps were followed by monitoring the absorbance at 280 nm. (B) The inset shows SDS-PAGE analysis of lysate sample [*S*], flow-through [*F*], wash [*W*] and elution [*E*] peak fractions. Molecular weight marker [*M*] was run in parallel. The purity was estimated by image analysis of SDS-polyacrylamide gels: the 43kDa recombinant protein is > 90% of the total protein content. The final yield was estimated at around 10 mg per liter of bacterial culture. (C) Identity of purified protein was checked by MALDI-TOF-TOF Mass Spectrometry. Peptide Mass Fingerprinting analysis showed a profile with 7 peptide tryptic masses matching with the theoretical masses originated by *in silico* digestion of human PDX1 sequence.

Recombinant Pdx1 fusion protein, containing a Protein Transduction Domain (PTD) of 16aa (R188-K203) allowing cell internalization, was obtained by linking 6His-tag to the N-terminus of amino acid sequence (S1 Fig). A 6His-tagged form of Pdx1 protein was generated by following the protocol developed by Koya *et al*. [19]. Heterologous expression in bacterial cells was optimized by providing a nucleotide sequence adapted to codon usage of *E*. *coli* and setting up conditions of growth after induction of recombinant protein (S2 Fig). Pdx1 was purified to homogeneity by using immobilized ion affinity chromatography followed by a second gel filtration chromatography step (Fig 1A and 1B). The overall procedure yielded 10 mg of Pdx1 protein per liter of culture. Protein purity was assessed as >90% based on densitometry (Fig 1B). The identity of the purified protein was confirmed by MALDI-TOF-TOF Mass Spectrometry. Peptide Mass Fingerprinting analysis showed a profile with 7 peptide masses matching the theoretical masses originated by *in silico* digestion of human Pdx1 sequence (Fig 1C). Moreover, peptide sequence determination obtained by MS/MS isolation and fragmentation of two tryptic peptides further confirmed the identity of the protein (data not shown).

### Cell internalization of Pdx1 peptide

**Fig 2:**
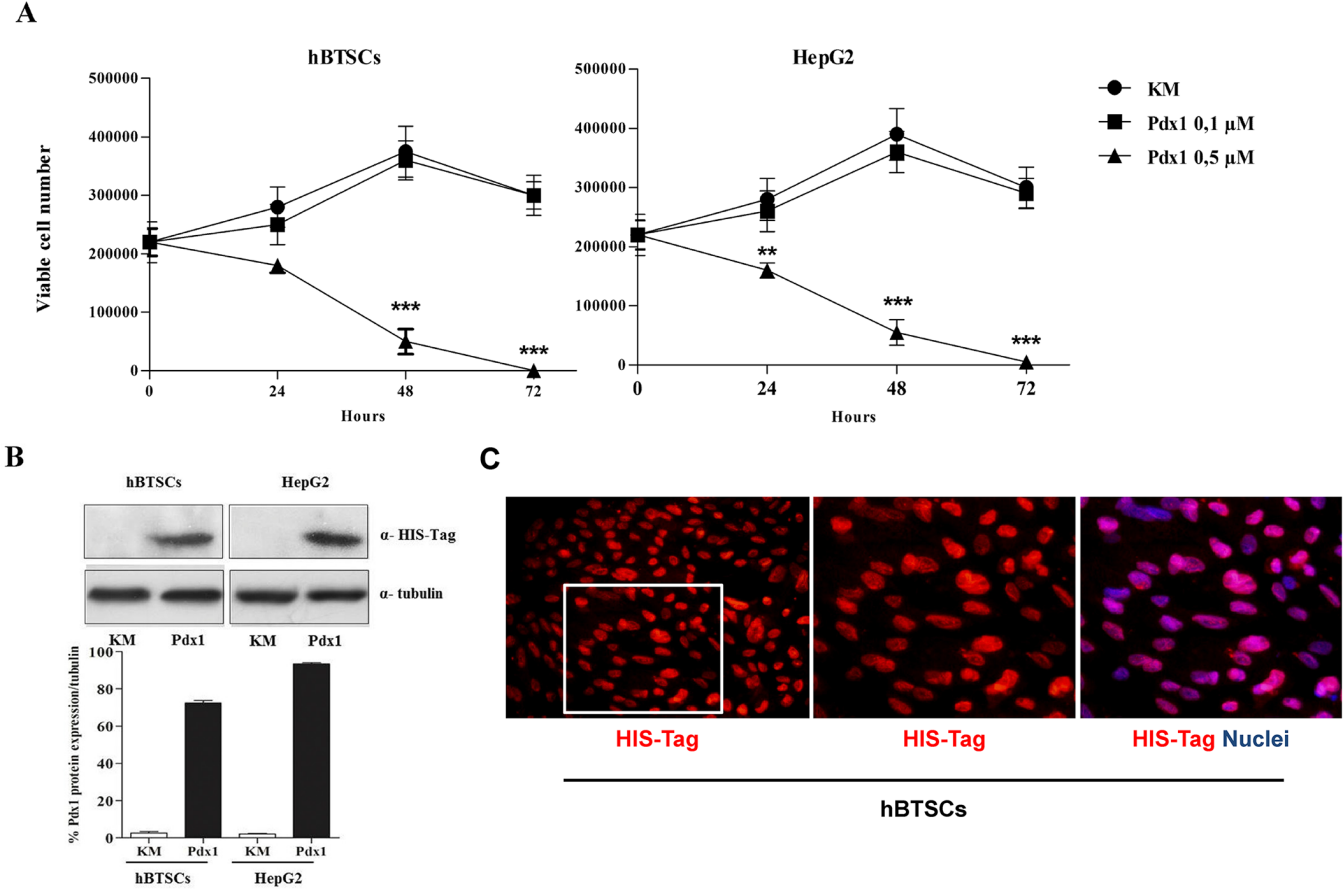
Pdx1 effects on cell viability and Pdx1 internalization. (A) hBTSCs and HepG2 were exposed to different concentrations of Pdx1 (0.1 μM or 0.5 μM). Incubation with 0.1 μM Pdx1 determined the preservation of cell viability. When cells were treated with 0.5 μM Pdx1, cell viability significantly decreased after 48 hours in both hBTSCs and HepG2 as observed by Trypan blue exclusion assay (data are means ± SD of 6 experiments; **p< 0.01; ***p< 0.001). (B) After 24 hours of treatment with 0.1 μM Pdx1 recombinant protein, Western Blot analyses showed the presence of Pdx1 recombinant protein (His-Tagged protein) in treated hBTSCs and HepG2. The densitometry histograms showed an equal amount of His-Tagged protein in hBTSCs and HepG2 cells treated with 0.1 μM Pdx1 (data are means ± SD of 3 experiments). (C) Immunofluorescence analysis showed the internalization and nuclear translocation of His-Tagged recombinant protein (red nuclei) in treated cells. Nuclei are displayed in blue (DAPI). Original Magnification = 20X left image or 40X right images.

First, we analyzed the effect of Pdx1 treatment on cell viability. Incubation with 0.1 μM Pdx1 for 3 days maintained cell viability (Fig 2A) and cell confluence (averaged 90%) both in hBTSCs and HepG2 cells. Conversely, the treatment with 0.5 μM Pdx1 determined a significant decrease of cell viability already after 48 h (p< 0.01) (Fig 2A). Therefore, the subsequent experiments were conducted by using the concentration of 0.1 μM Pdx1.

W-B analysis showed the presence of Pdx1 recombinant protein (His-Tagged protein) in cells treated for 24 hours with Pdx1 (Fig 2B). The Pdx1 uptake was comparable between hBTSCs and HepG2 cells as shown by densitometry analysis (Fig 2B). IF demonstrated the nuclear localization of the exogenous Pdx1; cellular internalization and nuclear localization of His-Tagged recombinant protein were evidenced in both hBTSCs (Fig 2C) and HepG2 cells (data not shown). These results showed safe and effective nuclear positioning of the recombinant Pdx1 at 0.1 μM concentration.

### Pdx1 peptide induced efficient *in vitro* differentiation of hBTSCs toward pancreatic islet cells

**Fig 3:**
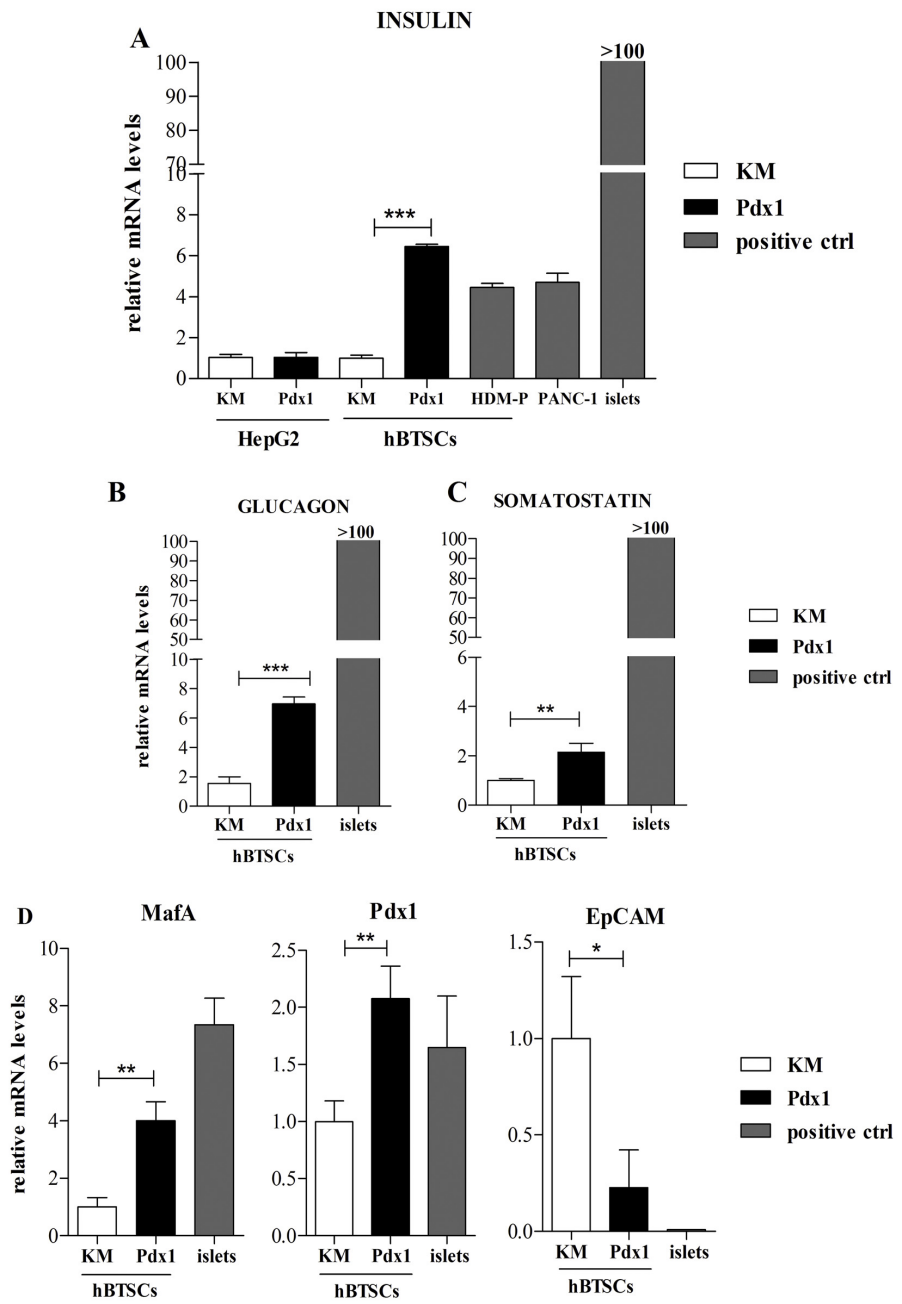
Human biliary tree stem cell (hBTSC) differentiation towards pancreatic fate induced *in vitro* by Pdx1 peptide. (A) In HepG2 cells the insulin mRNA level was not affected by 14 days of 0.1 μM Pdx1 administration, while it increased significantly (average 6 folds) in hBTSCs cultured for 14 days in Kubota’s Medium (KM) containing 0.1 μM Pdx1in comparison with control medium (KM) (data are means ± SD of 6 experiments; ***p< 0.001). The effect of Pdx1 administration on hBTSCs was similar to treatment with a Hormonally Defined Medium for Pancreatic islet cell differentiation (HDM-P). PANC-1 cells and pancreatic islets (islets) were used as positive controls. (B, C) In hBTSCs cultured for 14 days in KM containing 0.1 μM Pdx1, the glucagon and somatostatin mRNA levels increased 6- (data are means ± SD of 6 experiments; ***p< 0.001) and 2-folds (data are means ± SD of 6 experiments; **p< 0.01), respectively, in comparison with KM. (D) In hBTSCs cultured for 14 days in KM containing 0.1 μM Pdx1, MafA (4.5-folds) (data are means ± SD of 6 experiments; **p< 0.01) or Pdx1 (2-folds) (data are means ± SD of 6 experiments; **p< 0.01) gene expression increased in comparison with KM, while EpCAM mRNA decreased (average 4-folds) (data are means ± SD of 6 experiments; *p< 0.05).

Insulin mRNA expression was analyzed in hBTSCs and HepG2 cells treated with Pdx1 recombinant peptide; human pancreatic islets were used as controls. RT-PCR analyses (Fig 3A) demonstrated an increased expression of insulin mRNA in hBTSCs cultured for 14 days in medium containing 0.1 μM Pdx1 in comparison with hBTSCs maintained in control medium (KM) (p< 0.01). As shown in Fig 3A, after stimulation with 0.1 μM Pdx1, insulin mRNA expression in hBTSCs increased more than 6 fold with respect to control medium (KM) (p< 0.01) and showed results similar to hBTSCs cultured in HDM-P (4 fold induction versus KM). No difference in insulin mRNA expression was observed between Pdx1-stimulated hBTSCs and PANC-1 cells (Fig 3A). Insulin mRNA expression in normal human pancreatic islets resulted significantly higher in comparison with stimulated-(by Pdx1 or HDM-P)-hBTSCs or PANC-1 cells (p< 0.01). No induction of insulin mRNA expression was seen in HepG2 cells treated with 0.1 μM Pdx1 with respect to control medium (KM) (p< 0.05) (Fig 3A). The expression of Glucagon (Fig 3B) and somatostatin (Fig 3C) mRNA significantly increased, by 6- (p< 0.01) and 2-folds (p< 0.05) respectively, in hBTSCs treated for 14 days with 0.1 μM Pdx1with respect to KM alone. No effect of 0.1 μM Pdx1 on glucagon or somatostatin mRNA expression was seen in HepG2 cells with respect to control medium (KM) (data not shown). As observed for insulin expression, normal human pancreatic islets showed higher mRNA levels of glucagon (p< 0.01) (Fig 3B), and somatostatin (p< 0.01) (Fig 3C) with respect to hBTSCs treated with Pdx1. No differences in the expression of pancreatic islet markers have been observed between Pdx1-stimulated hBTSCs analyzed at day 3 (see above) versus day 30 after the last Pdx1 administration (data not shown), indicating the maintenance of Pdx1 effects for at least 30 days. We next evaluated the effects of Pdx1 treatment on pancreatic differentiation markers. The mRNA levels of pancreatic transcription factors MafA and Pdx1 were measured together with the epithelial stem cell marker, EpCAM. As shown in Fig 3D, a significant increase of MafA (p< 0.01) and Pdx1 (p< 0.01) mRNA was seen in hBTSCs stimulated with 0.1 μM Pdx1 for 14 days with respect to control medium (KM) while EpCAM gene expression decreased (p< 0.01).

### Morphological and functional characterization of the Pdx1 induced hBTSC-derived β-pancreatic islets

**Fig 4:**
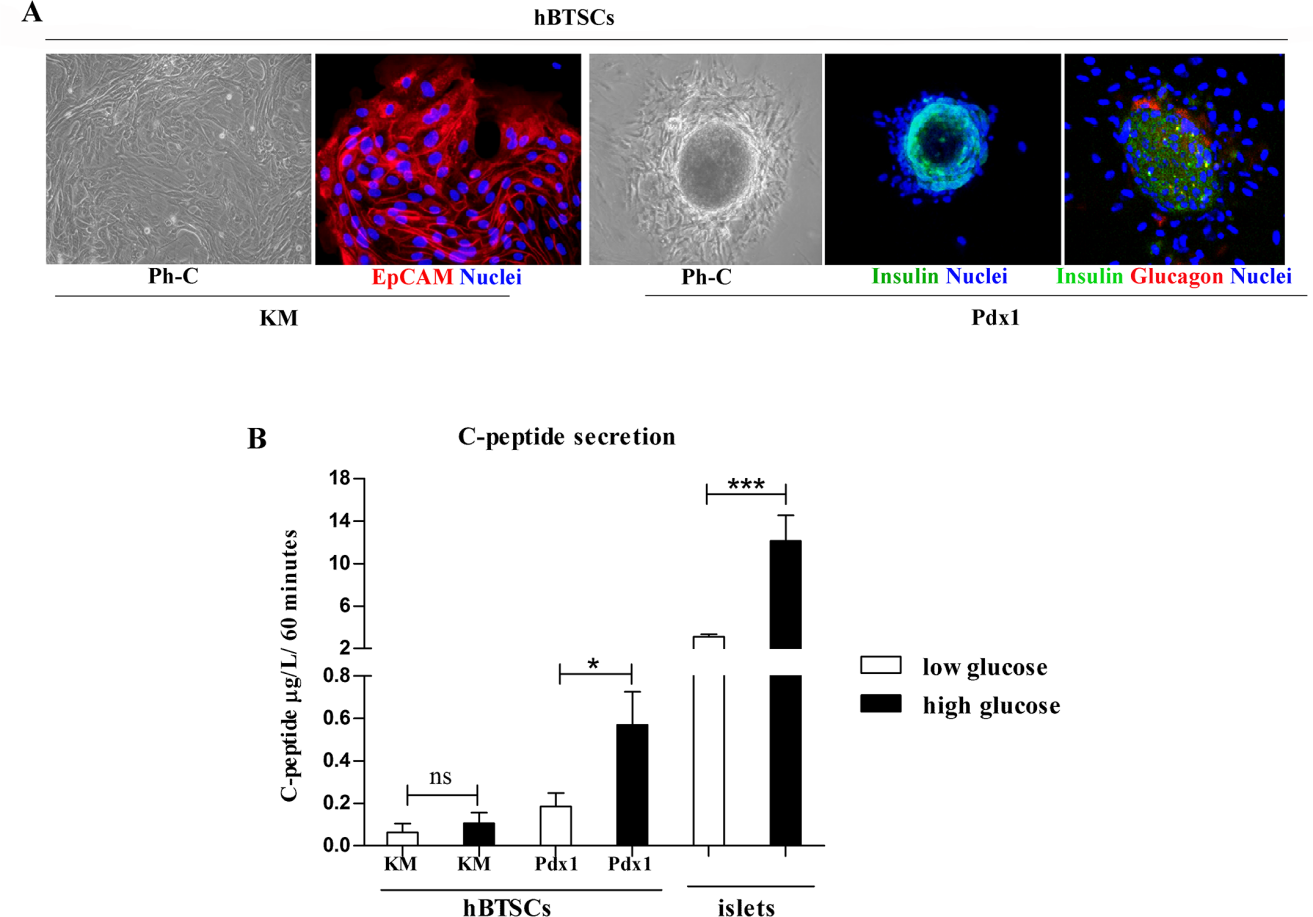
Pdx1 induced hBTSC-derived β-pancreatic islets are functioning active. (A) Morphologically (Phase Contrast: Ph-C; Original Magnification = 10X), after 14 days in basal condition, hBTSCs formed large colonies composed of small, densely packed, and uniform EpCAM-positive cells (Immunofluorescence for EpCAM; Original Magnification = 20X). After 14 days in cultures containing 0.1 μM Pdx1, several islet-like structures were present (Phase Contrast: Ph-C; Original Magnification = 10X); these structures were mostly composed of insulin-positive cells (Immunofluorescence for Insulin; Original Magnification = 10X); and some glucagon-positive cells were located at the periphery of these islet-like structures (double immunofluorescence for Insulin and Glucagon; Original Magnification = 20X). (B) Functionally, hBTSCs in Kubota’s Medium (KM) or in KM plus 0.1 μM Pdx1 were exposed to low (5.5 mM) or high (28 mM) glucose concentrations to stimulate C-peptide secretion and the response was compared with human pancreatic islets (islets). Human C-peptide secretion in hBTSCs in KM was not affected by high glucose stimulation (data are means ± SD of 6 experiments). In hBTSCs cultured for 14 days in KM containing 0.1 μM Pdx1, C-peptide secretion was detected at low glucose levels and further increased after exposure to high glucose concentration (data are means ± SD of 6 experiments; *p< 0.05). Human pancreatic islets (islets) were used as a positive control of the high glucose challenge (data are means ± SD of 6 experiments; *p< 0.05).

Very importantly, several islet-like structures (more than 5 for each chamber slide) were observed when hBTSCs were stimulated for 14 days with 0.1 μM Pdx1 (p< 0.01 vs KM; Fig 4A), and this was similar to hBTSCs cultured in HDM-P. These Pdx1-induced neoislets were composed of packed cells positive for insulin and glucagon staining (Fig 4A). In contrast, islet-like structures were not observed in hBTSCs maintained in control medium (KM) (Fig 4A). Finally, to evaluate the effective functional differentiation toward endocrine pancreatic cells, we analyzed, by ELISA, C-peptide secretion after challenging hBTSCs with high glucose concentration. Human pancreatic islets (islets) were used as a positive control. hBTSCs cultured for 14 days in 0.1 μM Pdx1 (n = 7) were exposed to low (5.5 mM) versus high (28 mM) glucose concentrations to stimulate C-peptide secretion (Fig 4B). hBTSCs cultured in KM failed to secrete C-peptide after glucose-challenge. In contrast, in hBTSCs treated with Pdx1 for 14 days, human C-peptide secretion in the medium at low glucose (0.22 ± 0.036 ug/L/60 mins) triplicated when cells were challenged with high glucose concentration (0.6 ± 0.046 ug/L/60 mins; p< 0.05). The C-peptide-release stimulation index measured in Pdx1-stimulated hBTSCs was similar with respect to that calculated in normal human pancreatic islets (secretion index = 3.02 ± 0.1 vs 3.4 ± 0.1, respectively).

## Discussion

Altogether our results indicate that the newly synthesized recombinant human Pdx1 efficiently lineage restricted hBTSCs to functional β-pancreatic islet cells. The efficient production of active recombinant human Pdx1 peptide was achieved by cloning the entire sequence of the human PDX1 gene containing a functional sequence coding for a protein transduction domain. The protein was rendered easily retrievable by a terminal coil of poly histidines that efficiently tagged the protein. The identity of purified protein was demonstrated by MALDI-TOF-TOF Mass Spectrometry.

The main purpose of our study was to evaluate the capability of recombinant Pdx1 to induce the differentiation of a population of normal hBTSCs toward β-pancreatic cells [6–11]. While cell internalization of Pdx1 was observed in both hBTSCs and HepG2 cells, the appearance of insulin producing functional neoislets was evident only in hBTSCs. Demonstration of equal quantities of Pdx1 uptake and appropriate localization of Pdx1 in hBTSCs and HepG2 cells have been obtained by both WB and IF. This excludes the possibility that failure of Pdx1 to induce HepG2 cell pancreatic differentiation was caused by its impaired uptake. Binding of the Pdx1 peptide to the chromatin has been only indirectly evaluated by nuclear staining. However, all the biological effects observed after exogenous Pdx1 administration can be effectively determined by Pdx1 per se since they were entirely compatible with the role of this transcription factor in inducing the differentiation of endodermal stem cells toward pancreatic fate [20]. The effective induction of insulin production by Pdx1 in hBTSCs was demonstrated by the marked increase of insulin-mRNA (6 times versus control hBTSCs), the appearance of insulin positive neo-islets (IF) and, finally, by the C-peptide secretion in the culture medium (ELISA). The observation of a parallel increase in glucagon and somatostatin further sustain the active role of Pdx1 in inducing endocrine differentiation of hBTSCs. Notably, further evidence of a genuine pro-differentiating role of the recombinant Pdx1 in hBTSCs arise from the parallel increase of pancreatic islet transcriptional factors (MafA, Pdx1), and the decrease of the endodermal stem cell marker EpCAM. Pdx1-induced neoislets were functionally active, since they responded to high glucose concentration triplicating C-peptide secretion and this response was similar to the one observed in human pancreatic islets. Moreover, the capability of Pdx1 peptide to induce pancreatic differentiation of hBTSCs was comparable to what observed when these cells were cultured in a medium specifically tailored for pancreatic differentiation (i.e. HDM-P medium) (1–6). When compared to human pancreatic islets, the Pdx1-stimulated hBTSCs showed lower expression of insulin and C-peptide secretion, indicating incomplete differentiation toward fully mature pancreatic islets. However, the hBTSC population is heterogeneous and this could justify a heterogeneous response of the different cell components. Indeed, under self-replication conditions in hBTSC cultures, the predominant cell population showed a phenotype resembling endodermal stem cells (EpCAM+/K7+/LGR5+/SOX17+/PDX1+). A more restricted subpopulation of cells with pluripotency features (SOX2+/OCT4A+/NANOG+) was also present (<10%) [11].

The effect of Pdx1 peptide on selected hBTSC subpopulations will be the purpose of forthcoming studies. In the last years, several attempts to reprogram liver cells into pancreatic endocrine cells have been proposed [1–3]. In our study, the failure of Pdx1 to induce pancreatic differentiation of mature hepatic HepG2 cells, despite the peptide internalization, is in keeping with recent investigations where early and late lineage stage liver parenchymal cells showed large differences in achieving pancreatic fate differentiation [4]. Pdx1 levels, in concert with Notch signalling, modulate the balanced differentiation of progenitor/stem cells versus endocrine pancreas rather than versus the biliary tree [20–25]. Pdx1 is observed in gallbladder and bile ducts (but not liver parenchyma) during embryonic development and in certain diseases [26, 27]. Defects in Notch signalling lead to accelerated pancreatic endocrine differentiation of bile duct cells driven by Pdx1 and Ngn3 [20–25]. Thus, hBTSCs retain, naturally, the factors potentially guiding pancreatic endocrine/exocrine differentiation. In the study by Banga et al. [5], Sox9 positivity, which marks progenitors, identified the cell types capable of being reprogrammed to pancreatic cells by the overexpression of three transcriptional factors (Pdx1, Ngn3 and MafA). The phenotypic traits and localization of these cells match that of hBTSCs [5–11, 21]. In our study, exogenous Pdx1 peptide induced over-expression of down-stream transcription factors (MafA), implicated in pancreatic endocrine specification. Thus, our results are consistent with data from developmental embryology and with the demonstration that Pdx1 positive cells occur naturally within PBGs throughout the biliary tree. Moreover, Pdx1 has been shown to play an active role during biliary pathologies associated with hBTSC proliferation [6–11, 21, 28, 29]. This indicates a neuroendocrine phenotype associated with biliary regenerative demand. Experiments in pre-clinical models of diabetes mellitus to assess *in situ* a Pdx1-mediated pancreatic islet differentiation of hBTSCs are needed. The demonstration that the recombinant human Pdx1 peptide alone exerted similar effects *in vitro*, as compared to the hormonally defined medium tailored to pancreatic islet fate, is particularly relevant in view of attempts to modulate the hBTSC compartment *in vivo*, where hormones and growth factors have limited applications because of their systemic effects. The selectivity of the Pdx1 effects observed in hBTSCs versus mature hepatocytes (HepG2) could facilitate the selective *in vivo* modulation of hBTSCs, *via* systemic Pdx1 administration. To this regard, the specific responsiveness of glandular elements of large intrahepatic bile ducts to pancreatic transcription factors administrated via systemic route has been already demonstrated in a mouse model [5]. The chromatin configuration of hBTSCs, in fact, could favor the access to pancreatic transcription factors and, their over-expression could be effective in cell reprogramming [5]. A pioneering strategy could be based on a selective delivery of Pdx1 to hBTSCs trough EpCAM-targeted nano-particles loaded with Pdx1.

## Supporting Information

S1 Fig PDX1 gene sequence. Full-length DNA coding sequence for human PDX1 (852 bp coding for 283 aa) adapted for heterologous expression in *E*. *coli* was provided by GenScript USA Inc. (Piscataway, NJ). Protein Transduction Domain (PTD), 16aa (R188-K203), allows cell internalization of the full protein. Recombinant Pdx1 was obtained in form of fusion protein by linking 6His-tag to the N-terminus of amino acid sequence.
(TIF)

S2 Fig Expression vector of PDX1 recombinant gene. After digestion with NdeI and BamHI, the amplicon was ligated into pET-28a expression vector (Novagen-Merck, Darmstadt, Germany), yielding pET-PDX1 plasmid. This construct was used to transform BL21 (DE3) *E*. *coli* strain (Invitrogen).
(TIF)
