# Supplementary figures and images for: Adult Human Biliary Tree Stem Cells Differentiate to β-Pancreatic Islet Cells by Treatment with a Recombinant Human Pdx1 Peptide
Source: PLoS One. 2015 Aug 7;10(8):e0134677. doi: 10.1371/journal.pone.0134677 (PMC4529196; doi:10.1371/journal.pone.0134677)

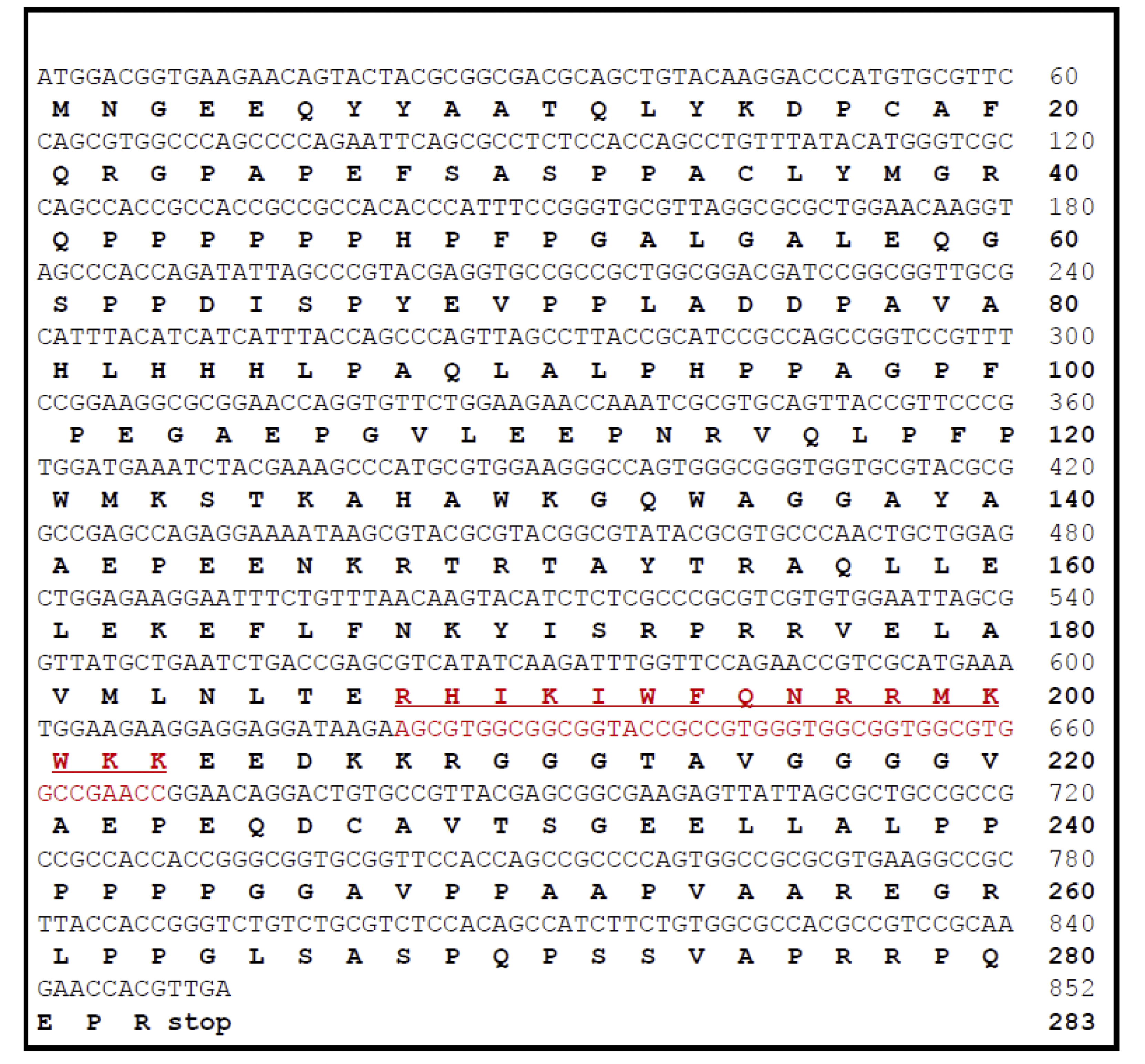

Supplement: S1 Fig — Full-length DNA coding sequence for human PDX1 (852 bp coding for 283 aa) adapted for heterologous expression in E. coli was provided by GenScript USA Inc. (Piscataway, NJ). Protein Transduction Domain (PTD), 16aa (R188-K203), allows cell internalization of the full protein. Recombinant Pdx1 was obtained in form of fusion protein by linking 6His-tag to the N-terminus of amino acid sequence. (TIF) [file pone.0134677.s001.tif]

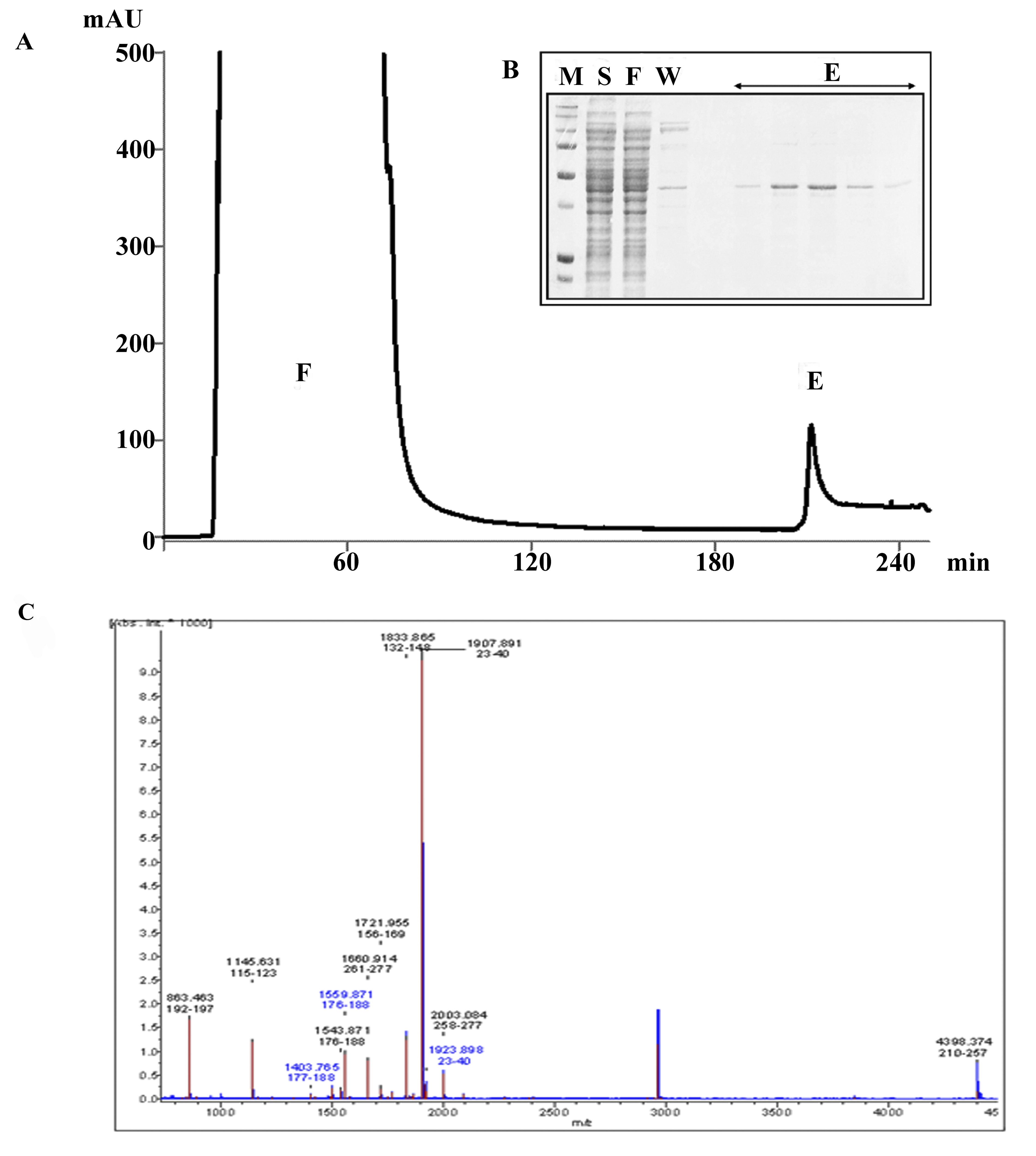

Supplement: S2 Fig — After digestion with NdeI and BamHI, the amplicon was ligated into pET-28a expression vector (Novagen-Merck, Darmstadt, Germany), yielding pET-PDX1 plasmid. This construct was used to transform BL21 (DE3) E. coli strain (Invitrogen). (TIF) [file pone.0134677.s002.tif]
